# A Simple-to-Use Nomogram for Predicting Postoperative Early Death Risk in Elderly Patients with Spinal Tumors: A Population-Based Study

**DOI:** 10.1155/2023/2805786

**Published:** 2023-03-04

**Authors:** Zhangheng Huang, Zhen Zhao, Yuheng Liu, Zhigang Zhou, Weifei Zhang, Qingquan Kong, Yaozhi He

**Affiliations:** ^1^Department of Orthopaedics, Orthopaedic Research Institute, West China Hospital, Sichuan University, Chengdu, Sichuan, China; ^2^Department of Orthopaedics, Jiujiang First People's Hospital, Jiujiang, Jiangxi, China; ^3^Department of Orthopaedics (Spine Surgery), The First Affiliated Hospital of Wenzhou Medical University, Wenzhou, Zhejiang, China

## Abstract

**Background:**

For elderly patients with primary spinal tumors, surgery is the best option for many elderly patients, in addition to palliative care. However, due to the unique physical function of elderly patients, the short-term prognosis is often unpredictable. It is therefore essential to develop a novel nomogram as a clinical aid to predict the risk of early death for elderly patients with primary spinal tumors who undergo surgery.

**Materials and Methods:**

In this study, clinical data were obtained from 651 patients through the SEER database, and they were retrospectively analyzed. Logistic regression analyses were used for risk-factor screening. Predictive modeling was performed through the R language. The prediction models were calibrated as well as evaluated for accuracy in the validation cohort. The receiver operating characteristic (ROC) curve and the decision curve analysis (DCA) were used to evaluate the functionality of the nomogram.

**Results:**

We identified four separate risk factors for constructing nomograms. The area under the receiver operating characteristic curve (training set 0.815, validation set 0.815) shows that the nomogram has good discrimination ability. The decision curve analysis demonstrates the clinical use of this nomogram. The calibration curve indicates that this nomogram has high accuracy. At the same time, we have also developed a web version of the online nomogram for clinical practitioners to apply.

**Conclusions:**

We have successfully developed a nomogram that can accurately predict the risk of early death of elderly patients with primary spinal tumors undergoing surgery, which can provide a reference for clinicians.

## 1. Introduction

Primary malignant tumors of the spine have an extremely low incidence as a rare bone malignancy. It accounts for approximately 5% of bone tumors and less than 0.2% of all malignant tumors [1, 2]. Osteosarcoma, chondrosarcoma, Ewing's sarcoma, and chordoma are the most common primary malignant tumors of the spine, accounting for approximately 35%, 30%, 16%, and 8.4%, respectively [1, 3]. Although the cause of some of these tumors is unclear (e.g., Ewing's sarcoma), neoadjuvant and adjuvant treatment with surgical resection has been adopted as the treatment of choice in the National Comprehensive Cancer Network (NCCN) guidelines and has been shown to have a 5-year survival rate of 66.6% [4]. However, due to the rarity of primary malignancies of the spine, studies have been conducted in almost all age groups. The elderly, as a population with a high prevalence of malignancy, have a higher risk of complications and a poorer prognosis after surgery due to deterioration in physical function and other underlying diseases [5, 6]. How to improve the prognosis of elderly patients with spinal malignancies after undergoing surgery is a challenge for clinical practitioners.

Nomograms are predictive models widely used by clinicians and have been applied to predict the prognosis of many types of cancer [7–10]. The Surveillance, Epidemiology, and End Results (SEER) database contains basic data on 28% of the US cancer patients, providing adequate and reliable sample for this study. Early death was considered to be death within six months of the initial surgery, and this definition has also been applied in other studies [11, 12]. To predict the early death in elderly patients with primary spinal tumors undergoing surgery, this nomogram was successfully constructed.

## 2. Materials and Methods

### 2.1. Patients and Data Collection

Patient data from the retrospective study came from the National Cancer Institute's Surveillance, Epidemiology, and End Results database (SEER) database, which covers cancer information for 28 percent of the total US population [13]. Because our study is retrospective and does not involve patient's personally identifiable information, ethical approval and patient consent are not required. We extracted clinical pathological data and demographic data from patients including age, race, sex, tumor grade, tumor stage, tumor histology, marital status, radiotherapy, chemotherapy status, and year of diagnosis.

The inclusion criteria were as follows: (1) the primary site was the spine, (2) a diagnosis of malignancy was made, and (3) the patient who was 60 years of age or older. Finally, 651 elderly patients diagnosed with primary malignant spinal tumors between 1975 and 2015 were included.

### 2.2. Statistical Analysis

The total information entries are randomly divided into a training cohort and a validation cohort at a ratio of 7 to 3 by using R software. Univariate logistic regression analysis was used to screen out variables in the training cohort that might affect the prognosis of elderly patients with primary spinal tumors undergoing surgery, and then, the above variables were screened again using multivariate logistic regression analysis to exclude confounding effects between different factors. Based on the risk factors screened by multivariate logistic regression analysis, we used R software to construct a nomogram. Using R software, the training cohort and the validation cohort are used to plot the receiver operating characteristic (ROC) curve, and the area under the curve (AUC) is closer to 1, indicating that the performance of this nomogram is better. Nomogram was evaluated for clinical efficacy using decision curve analysis (DCA). Calibration curves are used to assess the accuracy of predictions. All statistical analysis and charting were carried out by using SPSS (25.0) and R software version 4.0.3. The *p* value <0.05 is set to be statistically significant.

## 3. Results

### 3.1. Patients' Demographic Characteristics

Based on the inclusion criteria, a total of 651 elderly patients were included in this study. By the R software, all patients were randomized into a training cohort and a validation cohort in a 7 : 3 ratio. Detailed demographic and clinicopathological information of the above patients is presented in Table 1.

### 3.2. Associated Risk Factors

After univariate logistic regression analysis, age, tumor grade, tumor histology, and tumor stage became risk factors. Subsequently, the above variables were analyzed by multivariate logistic regression, and after removing the mixed factors between variables, it was determined that age, tumor grade, tumor histology, and tumor stage were all independent risk factors affecting the prognosis of elderly patients with primary spinal tumors who underwent surgery (Table 2).

### 3.3. Construction of Nomograms

**Figure 1:**
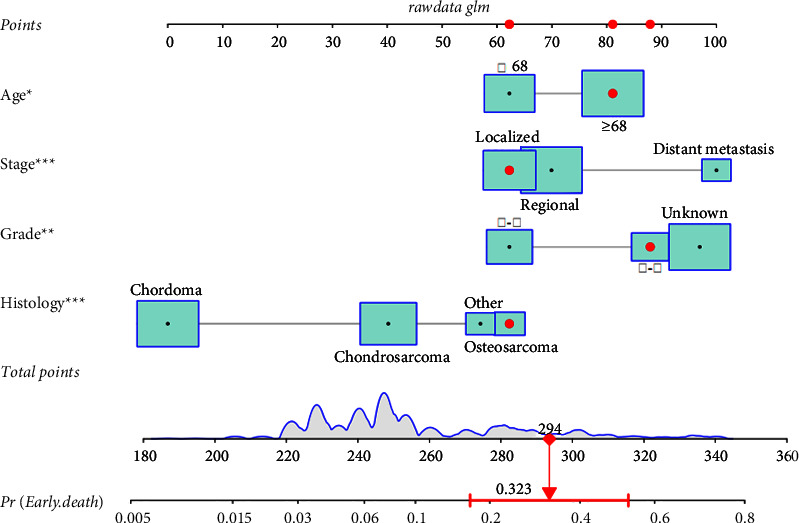
A nomogram for predicting the risk of early death in elderly patients with spinal tumors.

Nomograms that can predict the risk of early death in elderly patients with primary spinal tumors undergoing surgery are constructed based on four independent risk factors derived from the logistic regression analysis. The risk factors of the patients were corresponding to each point of the horizontal axis, and the relevant risk factors were scored separately. The prognostic prediction of the patient is obtained by adding the individual scores together to obtain a total score corresponding to the total score axis. We upload the nomograms that we have made and validated to the Internet so that they can be more easily used by more clinicians. The URL is https://hzhspine.shinyapps.io/hzhspine/ (Figure 1).

### 3.4. Evaluation of the Utility of Nomograms

**Figure 2:**
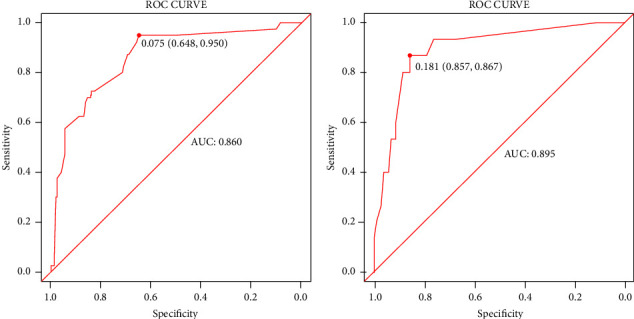
ROC curves for prediction of early-death risk in elderly patients with spinal tumors. The training cohort (a) and the validation cohort (b).

**Figure 3:**
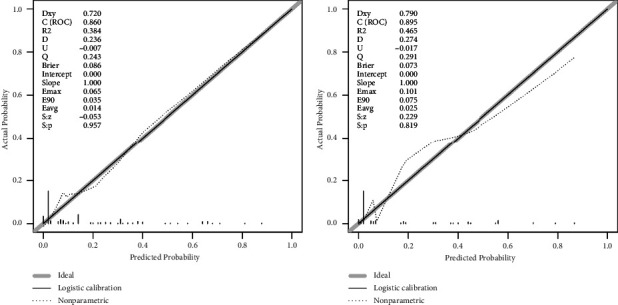
The calibration curves of the nomogram for predicting early-death risk in elderly patients with spinal tumors. The training cohort (a) and the validation cohort (b).

**Figure 4:**
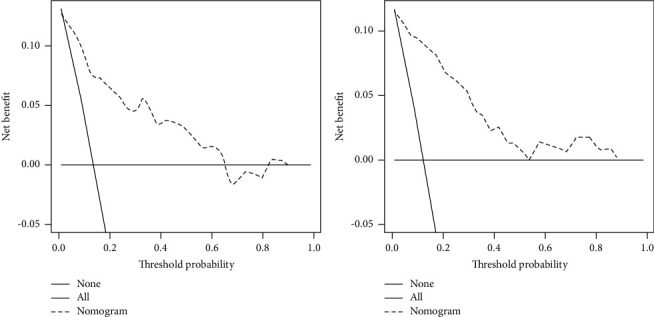
DCA of the nomogram for predicting the early-death risk in elderly patients with spinal tumors in the training cohort (a) and the validation cohort (b).

**Figure 5:**
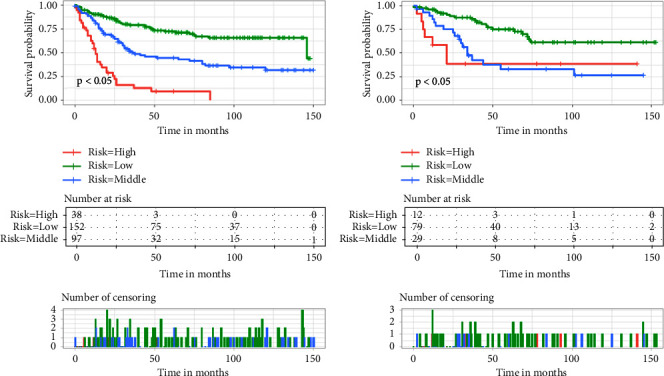
Kaplan-Meier survival curve for patients in different risk groups in both cohorts. The training cohort (a) and the validation cohort (b).

We evaluated this nomogram using the ROC curve and the DCA. The AUC of the ROC curve of the training and validation cohorts was 0.815 and 0.815, respectively, showing that the model had good differentiation (Figure 2). On the other hand, the correction curves of both the training and validation cohorts obtained an ideal curve close to 45°, demonstrating that the prediction tool has some accuracy (Figure 3). Decision-curve analysis shows that the nomogram can achieve an average good yield for patients under a certain risk threshold, which supports the clinical utility of this nomogram (Figure 4). Finally, we risk-stratified the cohort. The results of Kaplan–Meier test demonstrated significant variability between the different risk strata (Figure 5).

## 4. Discussion

Due to the rarity of primary spinal tumors, treatment strategies have only been progressively refined over the past two decades [14]. Even less attention has been paid to elderly patients. In the absence of relevant experience, reducing the risk of early death of elderly patients undergoing surgery for primary tumors of the spine is a major challenge for clinicians. Therefore, the development of a nomogram to predict the risk of early death of these elderly patients is of great clinical relevance. In this study, tumor grade, tumor stage, histological type of tumor, and patient's age were found to be statistically significant indicators that had a significant impact on patients' short-term prognosis. We can enter the above information for different individual patients into the nomogram to obtain an evaluation. It helps to take appropriate clinical measures according to patients' different risks of early death to improve prognosis.

It has been established that the occurrence of primary malignancies of the spine is significantly correlated with age, and that cancer mortality increases dramatically with age from the age of 65 years [15, 16]. This generally accepted view was also confirmed in the present study. In addition to the accumulation of exposure to predisposing factors, we believe that it is also related to the prevalence of underlying diseases in the elderly. The accumulation of these carcinogenic factors and the presence of underlying disease make the elderly less tolerant of adjuvant or neoadjuvant therapy, surgery, and postsurgical rehabilitation and also have a greater likelihood of tumor metastasis [17].

In addition to age, logistic regression analysis showed that tumor grade, tumor stage, and histological type of tumor are also key factors. The prognosis of survival in patients with primary tumors of the spine has been well-documented to correlate with these factors, and many authors have constructed related nomograms [10, 18–26]. In our previous studies, we have shown that tumor grade, tumor stage, and histological type of tumor are the key factors influencing postoperative OS in patients with primary spinal tumors, which is consistent with the results of this study [25]. However, almost all existing studies focus on all age groups, and no investigator has yet explored elderly patients as a study group.

Although in the present study, radiation therapy and chemotherapy were considered to be irrelevant factors for prognosis, and the view is consistent with many findings in the literature [10]. However, this still requires a different judgment on a case-by-case basis. In terms of tumor histology type, chondrosarcoma is resistant to radiation and chemotherapy, but multimodality therapy is still recommended in the treatment of Ewing's sarcoma and osteosarcoma [18, 23, 26–28]. In contrast, a subgroup analysis of the study by Zhou et al. showed that the malignant outcome associated with chemotherapy was instead mostly osteosarcoma and chondrosarcoma [29]. However, certainly, the application of radiotherapy and chemotherapy is closely related to the histological type of the tumor. On the other hand, it has also been pointed out that for spinal confined tumors, chemotherapy is one of the significant worsening influences, while for spinal metastatic tumors, chemotherapy has a good therapeutic effect [18, 29]. Unlike chemotherapy, radiotherapy is thought to have a significant tumor metastasis suppression effect, significantly reducing the probability of metastasis in the group of patients who receive radiotherapy immediately after surgery [30, 31].

Adequate presurgical evaluation is extremely important for elderly patients undergoing surgery for primary tumors of the spine [5]. The nomogram developed in this study was able to predict the risk of early death of surgery in these elderly patients. It has great clinical implications for assessing whether patients can tolerate the physical challenges associated with surgery and postoperative rehabilitation. We must acknowledge the limitations of this study, particularly the unavoidable problem of bias in retrospective studies. But this nomogram can also be used as a reliable preoperative assessment tool for risk reduction.

## 5. Conclusions

We have developed a nomogram to predict the risk of early death of elderly patients with primary spinal tumors undergoing surgery.

## Figures and Tables

**Table 1:** Demographic characteristics of elderly patients with spinal tumors.

Variables	Training cohort		Validation cohort	
	*N* = 459		*N* = 192	
	*n*	%	*n*	%
Age				
45–59	184	40.1	77	40.1
60–74	275	59.9	115	59.9
Race				
Black	12	2.6	7	3.6
White	422	91.9	177	92.2
Other	25	5.4	8	4.2
Sex				
Male	281	61.2	123	64.1
Female	178	38.8	69	35.9
Grade				
Grade I and II	132	28.8	52	27.1
Grade III and IV	88	19.1	38	19.8
Unknown	239	52.1	102	53.1
Stage				
Localized	171	37.3	70	36.5
Regional	235	51.2	103	53.6
Distant	53	11.5	19	9.9
Histological type				
Osteosarcoma	50	10.9	25	13.0
Chondrosarcoma	166	36.2	62	32.3
Chordoma	198	43.1	80	41.7
Other	45	9.9	25	13.0
Marital status				
No	143	31.1	52	27.1
Yes	316	68.9	140	72.9
Radiotherapy				
No	311	67.8	130	67.7
Yes	148	32.2	62	32.3
Chemotherapy				
No	412	89.8	169	88.0
Yes	47	10.2	23	12.0
Year of diagnosis				
1975–1984	35	7.6	14	7.3
1985–1994	72	15.7	26	13.5
1995–2004	100	21.8	58	30.2
2005–2015	252	54.9	96	50.0

**Table 2:** Results of univariate and multivariate logistic regression analysis in elderly patients with spinal tumors.

Characteristics	Univariate analysis		Multivariate analysis	
	HR (95% CI)	*p* value	HR (95% CI)	*p* value
Age				
45–59	Reference		Reference	
60–74	1.836 (1.041–3.240)	0.036	2.080 (1.107–3.907)	0.023
Race				
Black	Reference			
White	0.813 (0.174–3.802)	0.792		
Other	1.579 (0.268–9.308)	0.614		
Sex				
Male	Reference			
Female	1.077 (0.635–1.828)	0.783		
Grade				
Grade I and II	Reference		Reference	
Grade III and IV	4.125 (1.900–8.956)	<0.001	2.710 (1.140–6.442)	0.024
Unknown	1.700 (0.827–3.496)	0.149	3.852 (1.492–9.944)	0.005
Stage				
Localized	Reference		Reference	
Regional	1.310 (0.685–2.506)	0.414	1.346 (0.676–2.679)	0.398
Distant	7.427 (3.514–15.696)	<0.001	4.337 (1.901–9.893)	<0.001
Histological type				
Osteosarcoma	Reference		Reference	
Chondrosarcoma	0.236 (0.114–0.491)	<0.001	0.424 (0.182–0.987)	0.046
Chordoma	0.096 (0.042–0.221)	<0.001	0.089 (0.033–0.240)	<0.001
Other	0.900 (0.390–2.077)	0.805	0.814 (0.327–2.027)	0.659
Marital status				
No	Reference			
Yes	1.075 (0.611–1.891)	0.803		
Radiotherapy				
No	Reference			
Yes	0.807 (0.456–1.429)	0.462		
Chemotherapy				
No	Reference			
Yes	1.683 (0.793–3.572)	0.175		
Year of diagnosis				
1975–1984	Reference			
1985–1994	1.550 (0.461–5.207)	0.478		
1995–2004	1.937 (0.613–6.124)	0.260		
2005–2015	1.087 (0.359–3.289)	0.882		

## Data Availability

The publicly available datasets were analyzed in this study. These data can be found at the SEER dataset repository (https://seer.cancer.gov/).

## Abbreviations

SEER: Surveillance, Epidemiology, and End Results
OS: Overall survival
AUC: Area under the curve
DCA: Decision-curve analysis
